# Potassium [(1*S*)-1-azido-2-phenyl­eth­yl]trifluorido­borate

**DOI:** 10.1107/S1600536812030085

**Published:** 2012-07-10

**Authors:** Tore Lejon, Alexey S. Gorovoy, Victor N. Khrustalev

**Affiliations:** a Department of Chemistry, Faculty of Science and Technology, University of Tromsø, N-9037 Tromsø, Norway; b X-Ray Structural Centre, A.N. Nesmeyanov Institute of Organoelement Compounds, Russian Academy of Sciences, 28 Vavilov Street, B-334, Moscow 119991, Russian Federation

## Abstract

The title compound, K^+^·C_8_H_8_BF_3_N_3_^−^, is a salt containing the chiral organic trifluorido­borate anion. The organic anions and potassium cations are tightly bound to each other by the coordination K—F [2.654 (3)–3.102 (3) Å] and K—N [2.951 (4)–3.338 (4) Å] inter­actions. Thus, the potassium cation adopts a nine-vertex coordination polyhedron, which can be described as a distorted monocapped tetra­gonal anti­prism. In the crystal, the organic anions and potassium cations form layers parallel to (001). Weak C—H⋯π inter­actions between neighbouring phenyl rings further stabilize the crystal.

## Related literature

For the Matteson homologation, see: Matteson & Kim (2002 ▶); Matteson *et al.* (2006 ▶). For related compounds, see: Matteson & Beedle (1987 ▶); Scriven & Turnbull (1988 ▶); Darses & Genet (2008 ▶); Huang *et al.* (2009 ▶).
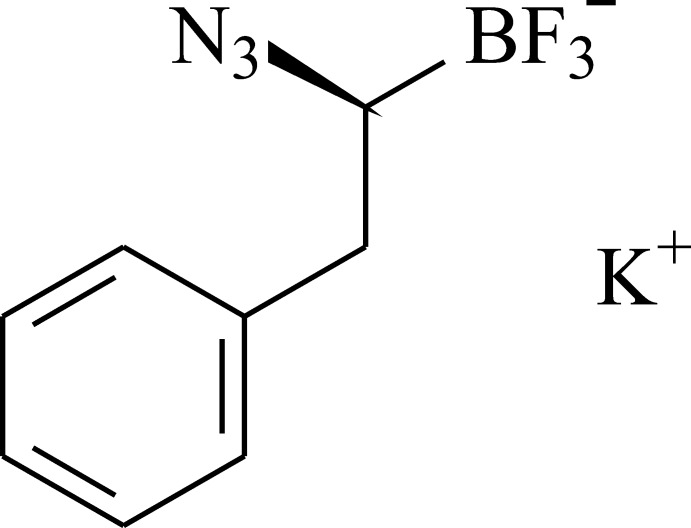


## Experimental

### 

#### Crystal data

K^+^·C_8_H_8_BF_3_N_3_^−^
*M* *_r_* = 253.08
Orthorhombic, 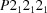

*a* = 6.052 (2) Å
*b* = 6.959 (2) Å
*c* = 25.120 (8) Å
*V* = 1057.9 (6) Å^3^
*Z* = 4
Mo *K*α radiation
μ = 0.52 mm^−1^
*T* = 100 K
0.15 × 0.10 × 0.01 mm

#### Data collection

Bruker APEXII CCD diffractometer
Absorption correction: multi-scan (*SADABS*; Sheldrick, 2003 ▶) *T*_min_ = 0.926, *T*_max_ = 0.995
10239 measured reflections
2075 independent reflections
1203 reflections with *I* > 2σ(*I*)
*R*_int_ = 0.060

#### Refinement

*R*[*F* ^2^ > 2σ(*F* ^2^)] = 0.050
*wR*(*F* ^2^) = 0.081
*S* = 0.98
2075 reflections
145 parameters
H-atom parameters constrained
Δρ_max_ = 0.33 e Å^−3^
Δρ_min_ = −0.40 e Å^−3^
Absolute structure: Flack (1983 ▶), 828 Friedel pairs
Flack parameter: 0.00 (8)

### 

Data collection: *APEX2* (Bruker, 2005 ▶); cell refinement: *SAINT* (Bruker, 2001 ▶); data reduction: *SAINT*; program(s) used to solve structure: *SHELXTL* (Sheldrick, 2008 ▶); program(s) used to refine structure: *SHELXTL*; molecular graphics: *SHELXTL*; software used to prepare material for publication: *SHELXTL*.

## Supplementary Material

Crystal structure: contains datablock(s) global, I. DOI: 10.1107/S1600536812030085/cv5318sup1.cif

Structure factors: contains datablock(s) I. DOI: 10.1107/S1600536812030085/cv5318Isup2.hkl

Additional supplementary materials:  crystallographic information; 3D view; checkCIF report

## Figures and Tables

**Table 1:** Weak C—H⋯π inter­actions between neighbouring phenyl rings (Å, °)

*D*—H⋯*A*	*D*—H	H⋯*A*	*D*⋯*A*	*D*—H⋯*A*
C4—H4⋯C3^i^	0.95	2.93	3.528 (6)	122
C4—H4⋯C4^i^	0.95	2.98	3.823 (6)	149
C4—H4⋯C5^i^	0.95	3.08	3.974 (7)	158
C4—H4⋯C6^i^	0.95	3.13	3.841 (7)	133
C4—H4⋯C7^i^	0.95	3.09	3.556 (7)	112
C4—H4⋯C8^i^	0.95	2.98	3.382 (6)	107

Symmetry code: (i) 
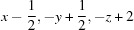
.

**Fig. 1.**
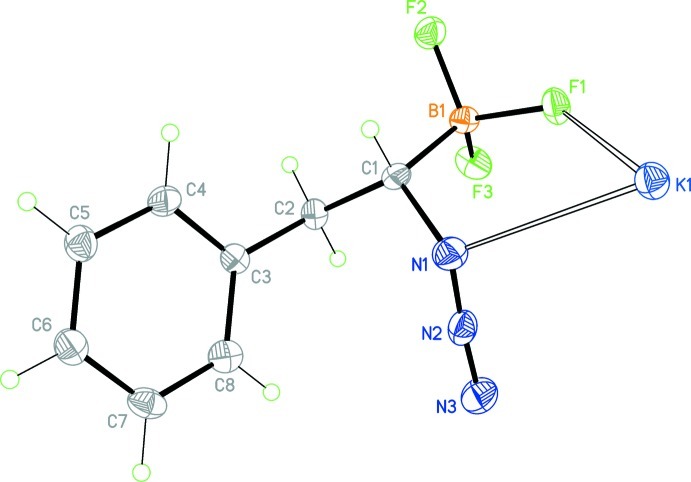
Independent part of the crystal structure of I. Displacement ellipsoids are shown at the 50% probability level.

**Fig. 2.**
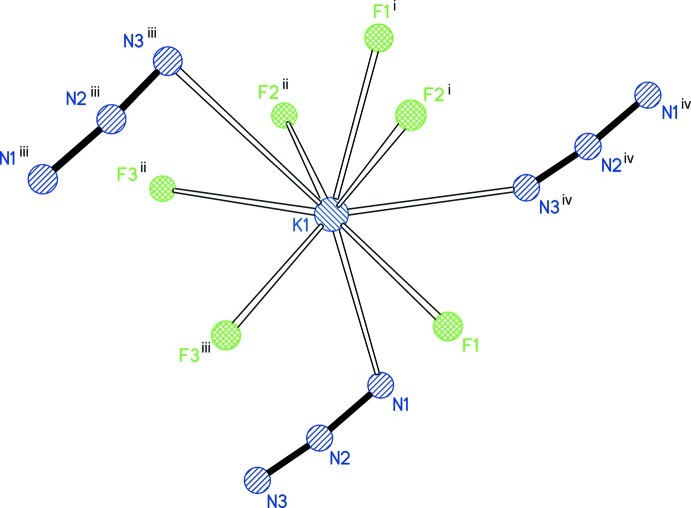
The coordination environment of potassium cation [symmetry codes: (i) -x, y + 1/2, -z + 3/2; (ii) x, y + 1, z; (iii) -x + 1, y + 1/2, -z + 3/2; (iv) x - 1, y + 1, z].

**Fig. 3.**
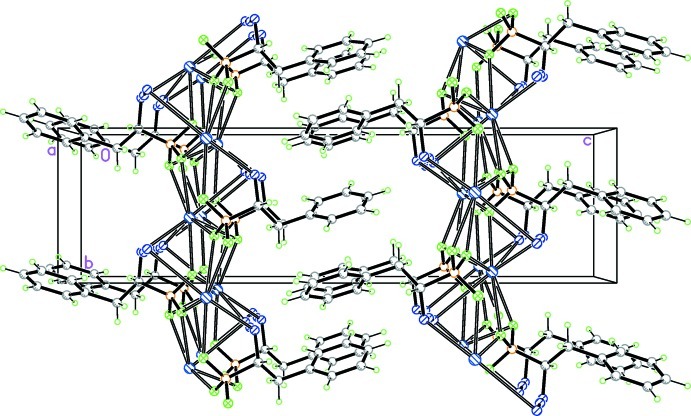
The layers in I parallel to (001).

**Fig. 4.**
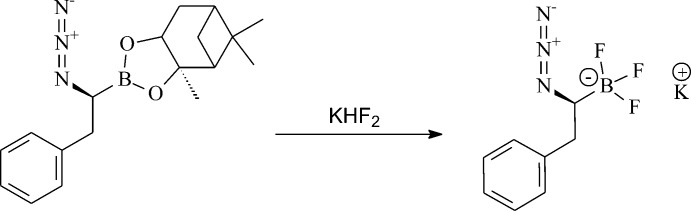
Reaction of 2-[(1S)-1-azido-2-phenyl-ethyl]-hexahydro-3a,5,5-trimethyl-4,6-methano-1,3,2-benzodioxaborole with potassium bifluoride.

## supplementary crystallographic information

### Comment

The target compound was synthesized as part of our studies on the Matteson homologation and the synthesis of chiral amino boronates. It was accomplished using potassium or caesium bifluorides which have been shown to be useful reagents in the deprotection of boronic esters (Matteson & Kim, 2002; Matteson *et al.*, 2006). However, organotrifluoridoborates are not only used for preservation and release of the boronic acid moiety, but also find application in different synthetic transformations (Darses & Genet, 2008). Moreover, organic azides have a rich chemistry and could prove important as building blocks in click chemistry (Scriven & Turnbull, 1988; Huang *et al.*, 2009).

The title compound - potassium [(1*S*)-1-azido-2-phenyl-ethyl]-trifluoro-boranuide, C_8_H_8_BF_3_KN_3_ (I), is a salt containing the chiral organic trifluoridoborate anion (Figure 1). It crystallizes in the orthorhombic space group *P*2_1_2_1_2_1_, with one crystallographically independent formula unit within the lattice cell. The asymmetrical C1 carbon atom has the *S*-configuration. The organic anions and potassium cations are tightly bound to each other by the coordination K—F [K1—F1 2.648 (3), K1—F1^i^ 2.673 (3), K1—F2^i^ 2.933 (3), K1—F2^ii^ 2.685 (3), K1—F3^ii^ 3.102 (3) and K1—F3^iii^ 2.654 (3) Å] and K—N [K1—N1 2.951 (4), K1—N3^iii^ 3.338 (4) and K1—N3^iv^ 3.020 (4) Å] interactions [symmetry codes: (i) -*x*, *y* + 1/2, -*z* + 3/2; (ii) *x*, *y* + 1, *z*; (iii) -*x* + 1, *y* + 1/2, -*z* + 3/2; (iv) *x* - 1, *y* + 1, *z*]. Thus, the potassium cation adopts the 9-vertex coordination polyhedron, which can be described as a distorted monocapped tetragonal antiprism (Figure 2).

In the crystal, the organic anions and potassium cations form the layers parallel to (001) (Figure 3). Moreover, the crystal packing is stabilized by the additional C4—H4···π interactions between the phenyl rings, with C4···Centroid^v^ distance of 3.418 (3) Å [symmetry code: (v) *x* - 1/2, -*y* + 1/2, -*z* + 2].

### Experimental

The initial 2-[(1*S*)-1-azido-2-phenyl-ethyl]-hexahydro-3a,5,5-trimethyl-4,6-methano-1,3,2-benzodioxaborole was obtained according to the procedure described earlier (Matteson & Beedle, 1987). A saturated water solution of potassium bifluoride (2.4 g, 30 mmol) was added drop wise to a solution of 2-[(1*S*)-1-azido-2-phenyl-ethyl]-hexahydro-3a,5,5-trimethyl-4,6-methano-1,3,2-benzodioxaborole (1.0 g, 3 mmol) in methanol (25 ml) at room temperature (Figure 4). The reaction mixture was stirred for 24 h. Then, the solvents were evaporated under reduced pressure (Attention! Hydrogen fluoride). The residue was washed with pentane and re-crystallized from hot methanol to give I as colourless crystals. Yield is 0.6 g (77%). ^1^H NMR (400 MHz, D_2_O, 293 K): δ = 7.57–7.12 (m, 5H), 2.94 (dd, *J* = 14.2, 3.3 Hz, 1H), 2.84 (m, 1H), 2.71 (m, 1H). ^13^C NMR (101 MHz, D_2_O, 293 K): δ = 141.68, 128.84, 128.51, 126.10, 35.55. ^19^F NMR (376 MHz, D_2_O, 293 K): δ = -145.40 (*s*).

### Refinement

The hydrogen atoms were placed in calculated positions with C—H = 0.95–1.00 Å and refined in the riding model with fixed isotropic displacement parameters [*U*_iso_(H) = 1.2*U*_eq_(C)].

### Crystal data

**Table:**

K^+^·C_8_H_8_BF_3_N_3_^−^	*F*(000) = 512
*M**_r_* = 253.08	*D*_x_ = 1.589 Mg m^−^^3^
Orthorhombic, *P*2_1_2_1_2_1_	Mo *K*α radiation, λ = 0.71073 Å
Hall symbol: P 2ac 2ab	Cell parameters from 546 reflections
*a* = 6.052 (2) Å	θ = 3.2–18.5°
*b* = 6.959 (2) Å	µ = 0.52 mm^−^^1^
*c* = 25.120 (8) Å	*T* = 100 K
*V* = 1057.9 (6) Å^3^	Plate, colourless
*Z* = 4	0.15 × 0.10 × 0.01 mm

### Data collection

**Table:**

Bruker APEXII CCD diffractometer	2075 independent reflections
Radiation source: fine-focus sealed tube	1203 reflections with *I* > 2σ(*I*)
Graphite monochromator	*R*_int_ = 0.060
φ and ω scans	θ_max_ = 26.0°, θ_min_ = 3.0°
Absorption correction: multi-scan (*SADABS*; Sheldrick, 2003)	*h* = −7→7
*T*_min_ = 0.926, *T*_max_ = 0.995	*k* = −8→8
10239 measured reflections	*l* = −30→30

### Refinement

**Table:**

Refinement on *F*^2^	Secondary atom site location: difference Fourier map
Least-squares matrix: full	Hydrogen site location: inferred from neighbouring sites
*R*[*F*^2^ > 2σ(*F*^2^)] = 0.050	H-atom parameters constrained
*wR*(*F*^2^) = 0.081	*w* = 1/[σ^2^(*F*_o_^2^) + (0.0191*P*)^2^] where *P* = (*F*_o_^2^ + 2*F*_c_^2^)/3
*S* = 0.98	(Δ/σ)_max_ < 0.001
2075 reflections	Δρ_max_ = 0.33 e Å^−^^3^
145 parameters	Δρ_min_ = −0.40 e Å^−^^3^
0 restraints	Absolute structure: Flack (1983), 828 Friedel pairs
Primary atom site location: structure-invariant direct methods	Flack parameter: 0.00 (8)

### Special details

**Table:**

Geometry. All e.s.d.'s (except the e.s.d. in the dihedral angle between two l.s. planes) are estimated using the full covariance matrix. The cell e.s.d.'s are taken into account individually in the estimation of e.s.d.'s in distances, angles and torsion angles; correlations between e.s.d.'s in cell parameters are only used when they are defined by crystal symmetry. An approximate (isotropic) treatment of cell e.s.d.'s is used for estimating e.s.d.'s involving l.s. planes.
Refinement. Refinement of *F*^2^ against ALL reflections. The weighted *R*-factor *wR* and goodness of fit *S* are based on *F*^2^, conventional *R*-factors *R* are based on *F*, with *F* set to zero for negative *F*^2^. The threshold expression of *F*^2^ > σ(*F*^2^) is used only for calculating *R*-factors(gt) *etc*. and is not relevant to the choice of reflections for refinement. *R*-factors based on *F*^2^ are statistically about twice as large as those based on *F*, and *R*- factors based on ALL data will be even larger.

### Fractional atomic coordinates and isotropic or equivalent isotropic displacement parameters (Å^2^)

**Table:**

	*x*	*y*	*z*	*U*_iso_*/*U*_eq_	
K1	0.19352 (19)	0.92096 (16)	0.77175 (5)	0.0230 (3)	
N1	0.4374 (7)	0.6992 (6)	0.85281 (14)	0.0243 (11)	
N2	0.6381 (7)	0.7035 (6)	0.84172 (15)	0.0224 (11)	
N3	0.8199 (7)	0.7267 (7)	0.83058 (14)	0.0296 (11)	
C1	0.3397 (8)	0.5009 (6)	0.86075 (17)	0.0187 (12)	
H1	0.1992	0.5194	0.8811	0.022*	
C2	0.4850 (8)	0.3714 (6)	0.89471 (16)	0.0206 (13)	
H2A	0.4156	0.2427	0.8965	0.025*	
H2B	0.6294	0.3564	0.8766	0.025*	
C3	0.5268 (8)	0.4407 (6)	0.95126 (18)	0.0167 (11)	
C4	0.3648 (8)	0.4141 (7)	0.99013 (17)	0.0215 (12)	
H4	0.2286	0.3546	0.9811	0.026*	
C5	0.4030 (8)	0.4748 (7)	1.04210 (19)	0.0227 (13)	
H5	0.2922	0.4575	1.0684	0.027*	
C6	0.6016 (8)	0.5603 (7)	1.05561 (19)	0.0231 (12)	
H6	0.6273	0.5998	1.0913	0.028*	
C7	0.7647 (8)	0.5888 (7)	1.01697 (18)	0.0236 (12)	
H7	0.9006	0.6489	1.0260	0.028*	
C8	0.7250 (8)	0.5283 (6)	0.96550 (18)	0.0219 (13)	
H8	0.8356	0.5469	0.9392	0.026*	
F1	0.1942 (4)	0.5408 (3)	0.76697 (10)	0.0241 (7)	
F2	0.0979 (4)	0.2693 (4)	0.81157 (9)	0.0250 (7)	
F3	0.4491 (4)	0.3061 (4)	0.78057 (9)	0.0270 (7)	
B1	0.2716 (9)	0.4042 (8)	0.8047 (2)	0.0171 (13)	

### Atomic displacement parameters (Å^2^)

**Table:**

	*U*^11^	*U*^22^	*U*^33^	*U*^12^	*U*^13^	*U*^23^
K1	0.0246 (6)	0.0215 (6)	0.0227 (6)	0.0008 (6)	−0.0020 (6)	0.0005 (6)
N1	0.021 (3)	0.028 (3)	0.024 (3)	−0.002 (2)	0.002 (2)	0.000 (2)
N2	0.031 (3)	0.017 (3)	0.019 (2)	−0.002 (2)	0.000 (2)	0.001 (2)
N3	0.025 (3)	0.036 (3)	0.029 (2)	−0.002 (3)	0.006 (2)	0.003 (2)
C1	0.017 (3)	0.017 (3)	0.022 (3)	−0.004 (2)	−0.001 (2)	0.004 (2)
C2	0.021 (3)	0.023 (3)	0.017 (3)	0.000 (2)	−0.001 (2)	0.003 (2)
C3	0.023 (3)	0.006 (3)	0.021 (3)	0.001 (2)	−0.005 (2)	0.002 (2)
C4	0.025 (3)	0.017 (3)	0.023 (3)	−0.006 (3)	−0.001 (2)	0.000 (3)
C5	0.025 (3)	0.025 (3)	0.019 (3)	0.001 (3)	0.001 (3)	−0.002 (2)
C6	0.030 (3)	0.017 (3)	0.022 (3)	−0.001 (3)	−0.004 (3)	0.001 (3)
C7	0.020 (3)	0.019 (3)	0.031 (3)	0.001 (3)	−0.007 (2)	0.000 (3)
C8	0.021 (3)	0.018 (3)	0.027 (3)	0.003 (2)	0.000 (3)	0.000 (2)
F1	0.0326 (17)	0.0189 (15)	0.0209 (16)	−0.0006 (15)	−0.0068 (16)	0.0024 (13)
F2	0.0288 (16)	0.0240 (17)	0.0221 (15)	−0.0069 (15)	−0.0006 (12)	0.0005 (15)
F3	0.0234 (16)	0.0341 (18)	0.0234 (16)	0.0050 (14)	0.0006 (13)	−0.0092 (15)
B1	0.017 (3)	0.013 (3)	0.021 (3)	0.000 (3)	0.000 (2)	−0.001 (3)

### Geometric parameters (Å, º)

**Table:**

K1—F1	2.648 (3)	C2—H2A	0.9900
K1—F3^i^	2.654 (3)	C2—H2B	0.9900
K1—F1^ii^	2.673 (3)	C3—C8	1.392 (6)
K1—F2^iii^	2.685 (3)	C3—C4	1.396 (6)
K1—F2^ii^	2.933 (3)	C4—C5	1.391 (6)
K1—N1	2.951 (4)	C4—H4	0.9500
K1—N3^iv^	3.020 (4)	C5—C6	1.383 (6)
K1—F3^iii^	3.102 (3)	C5—H5	0.9500
K1—N3^i^	3.338 (4)	C6—C7	1.399 (6)
N1—N2	1.246 (5)	C6—H6	0.9500
N1—C1	1.515 (6)	C7—C8	1.381 (6)
N2—N3	1.147 (5)	C7—H7	0.9500
C1—C2	1.521 (6)	C8—H8	0.9500
C1—B1	1.614 (7)	F1—B1	1.422 (6)
C1—H1	1.0000	F2—B1	1.420 (6)
C2—C3	1.521 (6)	F3—B1	1.410 (6)
			
F1—K1—F3^i^	71.05 (9)	N1—C1—B1	111.4 (4)
F1—K1—F1^ii^	107.25 (7)	C2—C1—B1	112.9 (4)
F3^i^—K1—F1^ii^	128.98 (8)	N1—C1—H1	106.4
F1—K1—F2^iii^	156.77 (10)	C2—C1—H1	106.4
F3^i^—K1—F2^iii^	129.20 (9)	B1—C1—H1	106.4
F1^ii^—K1—F2^iii^	70.42 (8)	C3—C2—C1	115.6 (4)
F1—K1—F2^ii^	66.99 (8)	C3—C2—H2A	108.4
F3^i^—K1—F2^ii^	91.61 (8)	C1—C2—H2A	108.4
F1^ii^—K1—F2^ii^	47.55 (7)	C3—C2—H2B	108.4
F2^iii^—K1—F2^ii^	117.46 (7)	C1—C2—H2B	108.4
F1—K1—N1	60.53 (10)	H2A—C2—H2B	107.4
F3^i^—K1—N1	77.08 (10)	C8—C3—C4	118.9 (4)
F1^ii^—K1—N1	148.57 (10)	C8—C3—C2	121.4 (4)
F2^iii^—K1—N1	108.83 (10)	C4—C3—C2	119.7 (4)
F2^ii^—K1—N1	127.22 (11)	C5—C4—C3	120.0 (4)
F1—K1—N3^iv^	64.93 (11)	C5—C4—H4	120.0
F3^i^—K1—N3^iv^	135.84 (11)	C3—C4—H4	120.0
F1^ii^—K1—N3^iv^	70.16 (10)	C6—C5—C4	120.3 (5)
F2^iii^—K1—N3^iv^	93.46 (11)	C6—C5—H5	119.8
F2^ii^—K1—N3^iv^	74.82 (10)	C4—C5—H5	119.8
N1—K1—N3^iv^	78.62 (11)	C5—C6—C7	120.3 (4)
F1—K1—F3^iii^	149.93 (9)	C5—C6—H6	119.9
F3^i^—K1—F3^iii^	83.65 (6)	C7—C6—H6	119.9
F1^ii^—K1—F3^iii^	101.20 (8)	C8—C7—C6	118.9 (4)
F2^iii^—K1—F3^iii^	45.66 (7)	C8—C7—H7	120.5
F2^ii^—K1—F3^iii^	131.42 (8)	C6—C7—H7	120.5
N1—K1—F3^iii^	98.81 (10)	C7—C8—C3	121.6 (5)
N3^iv^—K1—F3^iii^	136.46 (10)	C7—C8—H8	119.2
F1—K1—N3^i^	127.01 (11)	C3—C8—H8	119.2
F3^i^—K1—N3^i^	80.25 (10)	B1—F1—K1	129.7 (3)
F1^ii^—K1—N3^i^	59.97 (9)	B1—F1—K1^vii^	108.9 (3)
F2^iii^—K1—N3^i^	72.92 (9)	K1—F1—K1^vii^	109.06 (9)
F2^ii^—K1—N3^i^	70.44 (10)	B1—F2—K1^viii^	113.1 (3)
N1—K1—N3^i^	151.29 (11)	B1—F2—K1^vii^	96.9 (3)
N3^iv^—K1—N3^i^	130.08 (7)	K1^viii^—F2—K1^vii^	100.87 (8)
F3^iii^—K1—N3^i^	61.08 (9)	B1—F3—K1^vi^	133.5 (3)
N2—N1—C1	115.6 (4)	B1—F3—K1^viii^	94.0 (3)
N2—N1—K1	108.7 (3)	K1^vi^—F3—K1^viii^	129.14 (10)
C1—N1—K1	111.8 (3)	F3—B1—F2	107.2 (4)
N3—N2—N1	173.1 (5)	F3—B1—F1	106.7 (4)
N2—N3—K1^v^	154.2 (4)	F2—B1—F1	106.2 (4)
N2—N3—K1^vi^	94.3 (3)	F3—B1—C1	112.5 (4)
K1^v^—N3—K1^vi^	85.80 (9)	F2—B1—C1	111.0 (4)
N1—C1—C2	112.8 (4)	F1—B1—C1	112.7 (4)
			
F1—K1—N1—N2	101.9 (3)	N1—K1—F1—B1	9.8 (4)
F3^i^—K1—N1—N2	26.6 (3)	N3^iv^—K1—F1—B1	−81.1 (4)
F1^ii^—K1—N1—N2	176.1 (3)	F3^iii^—K1—F1—B1	61.1 (4)
F2^iii^—K1—N1—N2	−100.8 (3)	N3^i^—K1—F1—B1	156.4 (3)
F2^ii^—K1—N1—N2	108.6 (3)	F3^i^—K1—F1—K1^vii^	−127.74 (10)
N3^iv^—K1—N1—N2	169.4 (3)	F1^ii^—K1—F1—K1^vii^	−1.60 (5)
F3^iii^—K1—N1—N2	−54.8 (3)	F2^iii^—K1—F1—K1^vii^	78.8 (3)
N3^i^—K1—N1—N2	−12.2 (5)	F2^ii^—K1—F1—K1^vii^	−27.48 (8)
F1—K1—N1—C1	−26.9 (3)	N1—K1—F1—K1^vii^	146.70 (13)
F3^i^—K1—N1—C1	−102.3 (3)	N3^iv^—K1—F1—K1^vii^	55.89 (11)
F1^ii^—K1—N1—C1	47.3 (4)	F3^iii^—K1—F1—K1^vii^	−161.99 (14)
F2^iii^—K1—N1—C1	130.4 (3)	N3^i^—K1—F1—K1^vii^	−66.63 (15)
F2^ii^—K1—N1—C1	−20.2 (3)	K1^vi^—F3—B1—F2	161.9 (2)
N3^iv^—K1—N1—C1	40.6 (3)	K1^viii^—F3—B1—F2	2.3 (4)
F3^iii^—K1—N1—C1	176.4 (3)	K1^vi^—F3—B1—F1	48.4 (5)
N3^i^—K1—N1—C1	−141.0 (3)	K1^viii^—F3—B1—F1	−111.2 (3)
N2—N1—C1—C2	44.7 (5)	K1^vi^—F3—B1—C1	−75.8 (5)
K1—N1—C1—C2	169.7 (3)	K1^viii^—F3—B1—C1	124.6 (4)
N2—N1—C1—B1	−83.6 (5)	K1^viii^—F2—B1—F3	−2.8 (4)
K1—N1—C1—B1	41.5 (4)	K1^vii^—F2—B1—F3	−107.8 (3)
N1—C1—C2—C3	61.4 (5)	K1^viii^—F2—B1—F1	111.0 (3)
B1—C1—C2—C3	−171.2 (4)	K1^vii^—F2—B1—F1	6.1 (4)
C1—C2—C3—C8	−101.8 (5)	K1^viii^—F2—B1—C1	−126.1 (3)
C1—C2—C3—C4	79.0 (5)	K1^vii^—F2—B1—C1	128.9 (3)
C8—C3—C4—C5	0.0 (7)	K1—F1—B1—F3	−115.8 (3)
C2—C3—C4—C5	179.2 (4)	K1^vii^—F1—B1—F3	107.2 (3)
C3—C4—C5—C6	−0.5 (7)	K1—F1—B1—F2	130.0 (3)
C4—C5—C6—C7	0.9 (7)	K1^vii^—F1—B1—F2	−7.0 (4)
C5—C6—C7—C8	−0.8 (7)	K1—F1—B1—C1	8.2 (6)
C6—C7—C8—C3	0.3 (7)	K1^vii^—F1—B1—C1	−128.8 (3)
C4—C3—C8—C7	0.1 (7)	N1—C1—B1—F3	86.5 (5)
C2—C3—C8—C7	−179.0 (4)	C2—C1—B1—F3	−41.7 (6)
F3^i^—K1—F1—B1	95.3 (4)	N1—C1—B1—F2	−153.3 (4)
F1^ii^—K1—F1—B1	−138.5 (4)	C2—C1—B1—F2	78.5 (5)
F2^iii^—K1—F1—B1	−58.1 (5)	N1—C1—B1—F1	−34.3 (6)
F2^ii^—K1—F1—B1	−164.4 (4)	C2—C1—B1—F1	−162.5 (4)

Symmetry codes: (i) −*x*+1, *y*+1/2, −*z*+3/2; (ii) −*x*, *y*+1/2, −*z*+3/2; (iii) *x*, *y*+1, *z*; (iv) *x*−1, *y*, *z*; (v) *x*+1, *y*, *z*; (vi) −*x*+1, *y*−1/2, −*z*+3/2; (vii) −*x*, *y*−1/2, −*z*+3/2; (viii) *x*, *y*−1, *z*.

### Hydrogen-bond geometry (Å, º)

**Table:**

*D*—H···*A*	*D*—H	H···*A*	*D*···*A*	*D*—H···*A*
C4—H4···C3^ix^	0.95	2.93	3.528 (6)	122
C4—H4···C4^ix^	0.95	2.98	3.823 (6)	149
C4—H4···C5^ix^	0.95	3.08	3.974 (7)	158
C4—H4···C6^ix^	0.95	3.13	3.841 (7)	133
C4—H4···C7^ix^	0.95	3.09	3.556 (7)	112
C4—H4···C8^ix^	0.95	2.98	3.382 (6)	107

Symmetry code: (ix) *x*−1/2, −*y*+1/2, −*z*+2.
